# Individual and community empowerment improve resource users’ perceptions of community-based conservation effectiveness in Kenya and Tanzania

**DOI:** 10.1371/journal.pone.0301345

**Published:** 2024-04-30

**Authors:** Robert Y. Fidler, Shauna L. Mahajan, Lenice Ojwang, Samson Obiene, Tanguy Nicolas, Gabby N. Ahmadia, Lorna Slade, David O. Obura, Hope Beatty, Juma Mohamed, Joan Kawaka, Alastair R. Harborne

**Affiliations:** 1 Institute of Environment and Department of Biological Sciences, Florida International University, North Miami, Florida, United States of America; 2 Global Science, World Wildlife Fund US, Washington, District of Columbia, United States of America; 3 Ocean Conservation, World Wildlife Fund US, Washington, District of Columbia, United States of America; 4 Coastal Oceans Research and Development–Indian Ocean (CORDIO) East Africa, Mombasa, Kenya; 5 Fauna & Flora International, Cambridge, United Kingdom; 6 Mwambao Coastal Community Network Tanzania, Tanga, Tanzania; Wroclaw University of Environmental and Life Sciences: Uniwersytet Przyrodniczy we Wroclawiu, POLAND

## Abstract

Community-based conservation has been increasingly recognized as critical to achieve both conservation and socio-economic development goals worldwide. However, the long-term sustainability of community-based conservation programs is dependent on a broadly shared perception among community members that management actions are achieving their stated goals. Thus, understanding the underlying factors driving differences in perceptions of management effectiveness can help managers prioritize the processes and outcomes most valued by resource users and thereby promote sustained support for conservation efforts. Here, we utilize large-scale interview survey data and machine learning to identify the factors most strongly associated with differences in perceived management effectiveness between resource users engaged in marine community-based conservation programs in Kenya and Tanzania. Perceptions of management effectiveness were generally favorable in both countries, and the most important predictors of positive perceptions were associated with community and individual empowerment in resource management and use, but within disparate focal domains. Improved perceptions of management effectiveness in Kenya were closely related to increases in women’s empowerment in community-based conservation programs, while inclusionary and transparent governance structures were the most important factors driving improved perceptions in Tanzania. Additionally, the strongest predictors of differences between individuals in both countries often interacted synergistically to produce even higher rates of perceived effectiveness. These findings can help future initiatives in the region tailor management to match community-level priorities and emphasize the need for community-based conservation programs to understand local context to ensure that metrics of “success” are aligned with the needs and desires of local resource users.

## Introduction

Community-based conservation (CBC), which emphasizes the role of Indigenous peoples and local communities (IP and LCs) in natural resource management is increasingly recognized as critical for realizing joint biodiversity and sustainable development goals worldwide [1, 2]. The proliferation of CBC initiatives over the last decades [3, 4] is a response to the failures of “top-down” or “fortress” management, which has led to inequitably high socioeconomic impacts on local resource users [5]. Recent research shows that IP and LC-managed lands cover an estimated 40% of remaining intact natural areas [6], and have been degrading at slower rates than those under other forms of management [7], highlighting the increasing role of effective CBC initiatives for achieving sustainability goals worldwide.

The long-term sustainability of CBC programs depends on successful collective action [8], which requires active participation of IP and LCs at multiple levels of resource management and governance [9, 10]. Among the most important prerequisites for sustained local participation in CBC is a shared and continued perception that management actions are effective at achieving their stated goals [11]. Individuals’ perceptions of CBC effectiveness, namely the way in which they interpret and evaluate the policies and outcomes of management [12], are inherently subjective and can be influenced by a variety of factors, including local context, past experiences, and individual and group attributes [13, 14]. Thus, perceptions can vary significantly among individuals even when they possess accurate and similar prior knowledge.

Differences in perceptions of management effectiveness within communities engaged in CBC can have significant impacts on long-term conservation success. Local understanding of the importance of CBC and program outcomes can influence perceived costs and benefits for resource users [15] and their willingness to comply with CBC regulations [16], both of which have been shown to be critical for CBC efficacy. An increased sense of equity in the distribution of costs and benefits within CBC programs can improve individual- and community-level support for conservation [17–19], while participation in governance and community-level consultation of resource-users by managing bodies can increase perceived legitimacy of local management [20–22]. Therefore, examining what drives disparities in user perceptions of CBC efficacy can help managers understand what aspects of CBC initiatives are promoting or weakening support for conservation, whether certain management practices are likely to be supported by community members [23], and to prioritize actions that provide the most important benefits to resource users or mechanisms that make those benefits more apparent [24].

Among the most critically important geographies that have widely adopted CBC are coastal east African Regions bordering the Western Indian Ocean (WIO). The WIO is a global diversity hotspot [25, 26] containing ~16% of the world’s coral reefs [27], high levels of marine endemism [28] and strong reliance on coastal fisheries for local livelihoods, food security, and national economies [29]. To protect this highly important region, governments, conservation organizations, and community-level fisheries management bodies have come together to promote the effective governance and expansion of successful CBC, often realized through approaches such as fisheries co-management and locally managed marine areas [30, 31].

Individual and community perceptions of CBC effectiveness in Africa have been demonstrated to be impacted by myriad, context-specific factors across regions and conservation programs. Previous studies have indicated that perceptions of management efficacy can be mediated by the degree of awareness of conservation programs and types of interactions with managers [32], individual occupations and level of education [33], and whether individuals believe they are receiving tangible benefits from conservation programs and can actively participate in management, particularly in terms of empowerment to influence rule- and decision-making structures [24, 34]. These perceptions can be further modulated by individual demographic and personality characteristics, social norms and values, and cultural contexts specific to communities [35], necessitating that community- and project-based investigations into user perceptions of CBC programs be incorporated into management strategies [12]. Thus, understanding the factors that lead to more positive perceptions of management effectiveness across different geographical contexts in the WIO, and therefore higher community-level support for conservation programs, will be critical to the success of CBC initiatives throughout the region.

Here, we assess individual differences in perceived management effectiveness among community members associated with marine CBC initiatives in two countries in the WIO: Kenya and Tanzania. Using large-scale household survey data from both countries, we utilize machine learning to identify the factors that most accurately predict whether individuals are more or less likely to perceive local CBC management to be effective, assess whether those factors act in synergistic or antagonistic patterns to shape individual perceptions of management, and compare and contrast patterns of individual perceptions between the two countries.

## Materials and methods

### Research approach

**Fig 1:**
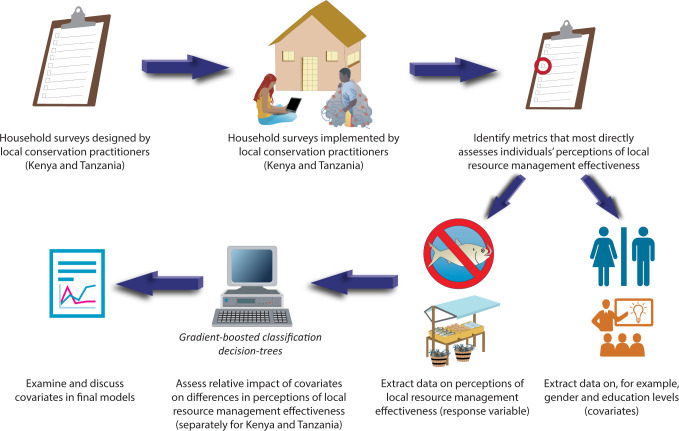
Schematic overview of the research approach utilized in this study.

This study leveraged existing household survey data collected to support marine CBC project design in both Kenya and Tanzania. Household surveys were designed and implemented by local conservation practitioners with intimate knowledge of local management structures and socio-economic contexts to meet ongoing conservation needs. From each survey instrument, we identified the metric that most directly assessed individuals’ perceptions of the effectiveness of local resource management, as well as a suite of covariates that could potentially impact those perceptions. After covariate selection, separate analytical models were built for each country to assess the relative impact of covariates on differences in perceptions of CBC efficacy. An overview of the research approach can be seen in Fig 1 and additional details of each step can be found in the following sections.

### Community-Based conservation at study locations

Efforts to advance marine conservation at study sites in both countries could be categorized as existing somewhere on the spectrum between “consultative” and “cooperative” co-management systems [36]. However, while similar in some respects, each system has nuanced distinctions in terms of membership and power dynamics between local management bodies and resource users as well as autonomy in decision-making from national governance structures.

#### Kenya

In Kenya, marine CBC focused on fisheries co-management is implemented through Beach Management Units (BMUs), introduced by the Ministry of Fisheries in 2006 [37]. A BMU is composed of local fishers and other stakeholders that are impacted by, and make decisions regarding, marine resource management. BMUs are defined by areas where fishers land their catches and are jurisdictionally limited to “inshore waters” along their designated coastlines. Within these regions, BMUs assist the Ministry in charge of fisheries in recording catches and enforcing regulations and can also develop their own by-laws and co-management plans, though final approval and endorsement of these frameworks rests with the government at the county and national level respectively.

#### Tanzania (Zanzibar)

In the United Republic of Tanzania, assessments were conducted on Pemba Island in Zanzibar, a semi-autonomous archipelago that lies 25–50 km off of the mainland. Due to the semi-autonomous status of Zanzibar, the practices and government regulations surrounding fisheries co-management and CBC in general are similar but distinct to those of mainland Tanzania. In Zanzibar, CBC takes the form of Shehia Fisheries Committees (SFCs; also known as Village Fisheries Committees [VFCs]), which were developed by the Zanzibar Department of Fisheries beginning in 1994. SFCs are community-led institutions that include 10 elected marine resource users and are guided by government fisheries departments. In support of the Department of Fisheries, SFCs oversee fisheries and enforce regulations, and can create by-laws regarding local marine resource use.

### Household surveys

Perceptions of CBC management effectiveness and associated covariates were derived from household surveys conducted in each country using similar but distinct survey instruments. Although both instruments were designed to capture a range of social and governance indicators related to CBC, the specific indicators measured by each instrument varied, even when assessing related governance principles (described below; see the S1 Appendix for a full list of metrics calculated for each instrument). We therefore analyze sites in Kenya and Tanzania separately to ensure that contextual factors unique to each country and instrument are adequately accounted for in the models.

#### Kenya

**Fig 2:**
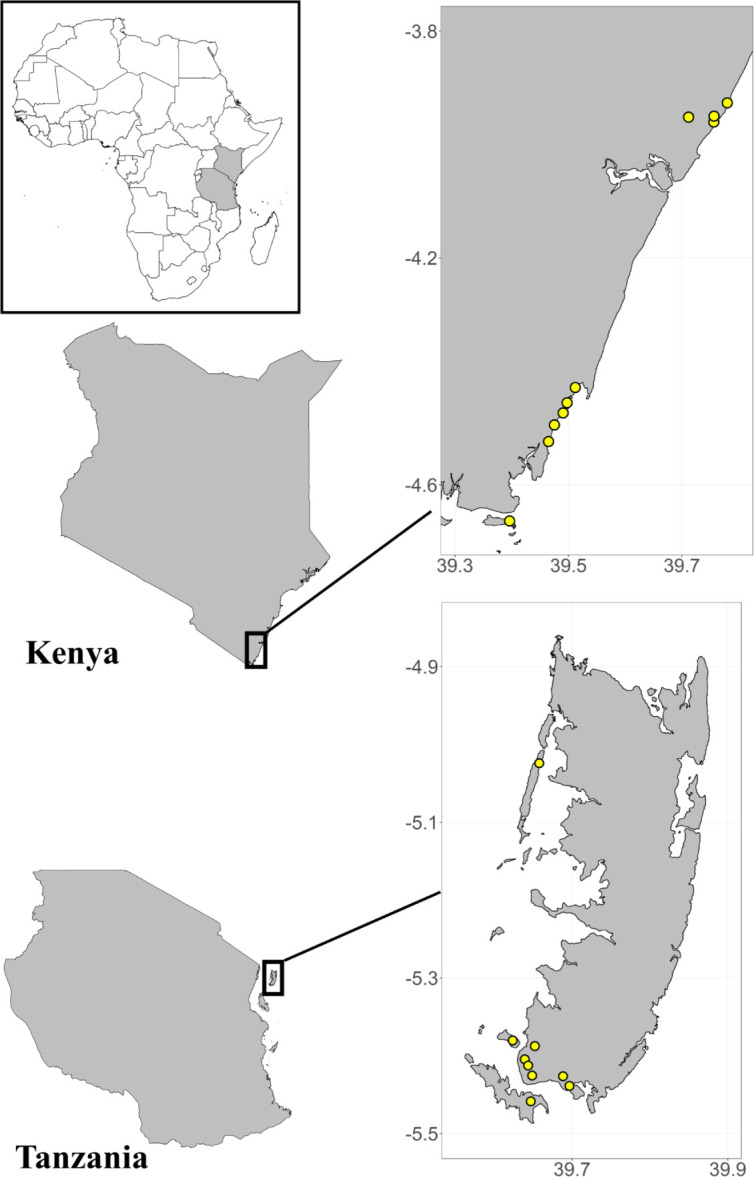
Location of household surveys in Kenya and Tanzania. Yellow circles indicate locations where household survey data was collected.

The status and impacts of CBC in Kenya were assessed using data collected from 406 households in 10 BMUs in 2021 (Fig 2). Households were selected for surveys using simple random sampling, and individuals within households were surveyed only if they were at least 18 years old, had lived predominantly in the study location for an extended period, and actively participated in marine-related activities. Sampling followed the design of Mugenda and Mugenda [38], which recommends surveying 10–30% of the study population. The survey instrument was designed to assess the awareness and perception of fishing communities of climate change, the adoption of climate smart practices, and the socioeconomic impacts of ongoing local CBC interventions. Questions in the survey included demographic characteristics (e.g., gender and education level), metrics of BMU membership and engagement in resource management (e.g., whether individuals were members of BMUs and their relative position and length of service within BMU management), reliance on marine resources for both income and food security (e.g., whether individuals were fishers or employed in related marine occupations), and the use and knowledge of destructive and sustainable fishing gears (S1 Table in S1 Appendix).

#### Tanzania (Zanzibar)

On Pemba Island, a total of 526 household surveys were conducted across nine SFCs associated with the Pemba Channel Conservation Area (PECCA) over nine consecutive days in 2021 (Fig 2). Surveys were conducted randomly within communities, aimed to sample enough individuals to achieve confidence levels of 90%, and achieve roughly equivalent representation of both genders. Sample sizes were determined based on 2012 census data and local population registries. Questions in the survey included demographic characteristics (e.g., gender and education), indicators of resource governance structures and individual participation in management (e.g., whether individuals were SFC members and their knowledge of SFC activities), reliance on marine resources for income and food security (e.g., whether individuals were fishers or employed in related marine occupations), and indicators of current and recent trends in overall wellbeing (S2 Table in S1 Appendix).

### Perceptions of management effectiveness and covariates

For each survey instrument, we identified the question that could most directly provide insight into individual perceptions of local CBC management while also being coded in a binary scale for analysis.

In Kenya, individual perceptions of governance effectiveness of CBC programs were derived from the question: “*Are you satisfied with how members of your group benefit from natural resource management*?*”*, to which respondents could respond “yes”, “no”, or “not sure.” Individuals that responded “yes” were considered to have perceived management as effective, and those that responded “no” to have perceived management as not effective. Surveys from individuals that responded “not sure” were removed, resulting in a total of 349 individuals.

In Tanzania, perceptions of CBC effectiveness were determined by responses to the question: *“Do you think the Shehia Fisheries Committee (SFC) is effective in the activities you believe to be its responsibilities*?”, to which respondents could respond “very effective”, “effective”, “not effective”, or “not sure.” Individuals that responded “very effective” or “effective” were considered to have perceived management as effective, and those that responded “not effective” to have perceived management as not effective. Surveys from individuals that responded “not sure” were removed, resulting in a total of 279 individuals.

In both countries, all other questions from each survey were included in analyses as covariates provided that: responses could be coded in either a Likert or binary scale, had at least two respondents for each possible response value for that question, and contained no more than 20% missing or “NA” values. After feature selection, a total of 45 and 49 predictive variables were included in models for Kenya and Tanzania, respectively.

### Model specification and analyses

We used gradient-boosted classification decision-trees to examine the most important features associated with patterns of perceived CBC effectiveness. Decision-tree models were utilized because they do not require data to fit the assumptions associated with parametric tests and are able to account for higher-order interactions, mixed data types, and nonlinear relationships between variables. Models were created with an extreme gradient boosting algorithm using the *xgboost* function in the *xgboost* package v1.6.0.1 [39] for R statistical software v4.2.1. [40]. For each country, surveys were split randomly into a training set (75% of data; Kenya: 261 surveys; Tanzania: 209 surveys) to specify the model and a testing set (25%; Kenya: 88 surveys; Tanzania: 70 surveys) to test model predictive accuracy.

The *xgboost* algorithm provides numerous options for model tuning to increase predictive accuracy. To determine ideal model specifications, we trained 9,000 iterations of each model using the training data with all possible combinations of five tuning parameters (Table 1). At each iteration, the model was applied to the testing set to check for predictive accuracy. The model with parameters that produced the highest predictive accuracy for each country was then run 100 times to account for variation in tree building and subsampling of features and data. Final predictive accuracy and relative feature importance are presented as the mean and standard deviation of all 100 model runs.

**Table 1:** Parameter values used in iterative optimization of classification models and final values used for each parameter for each country-specific model.

Parameter	Definition	Range Tested	Increments	Kenya	Tanzania
eta	Learning rate: shrinks feature weights after each round to reach the best optimum	0.02–0.3	0.02	0.18	0.28
max_depth	Maximum tree depth: model complexity	2–6	1	6	5
subsample	Subsamples: the number of observations supplied to a tree	0.25–0.75	0.25	0.50	0.25
colsample_bytree	Feature samples: the number of features supplied to a tree	1/3–2/3	1/3	2/3	2/3
scale_pos_weight	Positive weight scale: corrects for inbalances in response variable values	1.2–5	0.2	1.6	1.2

## Results

### Survey response data

In both countries, overall perceptions of management effectiveness were generally positive, with 67% (n = 349) and 81% (n = 279) of individuals responding that they believed management was effective in Kenya and Tanzania, respectively. Perceptions of management effectiveness were similar between genders, with 61% (n = 211) of males and 75% (n = 138) of females indicating that CBC was effective in Kenya, and 77% (n = 162) of males and 87% (n = 117) of females stating CBC was effective in Tanzania, although some site-specific variation did exist (Table 2). Covariate data from surveys was also robust for both countries. In Kenya, 27 of 45 metrics contained no “NA” data, and no individual question contained more than 5% “NA” responses. In Tanzania, 41 of 49 metrics contained no “NA” responses, and no individual question contained more than 18% “NA” values. Descriptions of all metrics and relative response rates for each possible value can be found in S1, S2 Tables in S1 Appendix.

**Table 2:** Gender-specific number of surveys (N) from each site in Kenya and Tanzania that were included in analyses. The “% Effective” column represents the number of female or male respondents from each site that indicated that they believed management was effective.

Kenya				Tanzania			
Site	Gender	N	% Effective	Site	Gender	N	% Effective
Gazi	Female	16	87.50	Chokocho	Female	11	72.73
	Male	18	44.44		Male	17	70.59
Kanamai	Female	14	71.43	Fundo	Female	14	92.86
	Male	13	76.92		Male	16	81.25
Kidongo	Female	12	91.67	Kangani	Female	16	75.00
	Male	13	92.31		Male	19	84.21
Marina	Female	7	14.29	Kisiwa Panza	Female	11	72.73
	Male	22	40.91		Male	18	66.67
Mkunguni	Female	13	76.92	Kukuu	Female	23	100.00
	Male	28	57.14		Male	24	100.00
Mkwiro	Female	22	77.27	Makoongwe	Female	17	88.24
	Male	20	70.00		Male	19	94.74
Mtwapa	Female	6	50.00	Michenzani	Female	3	66.67
	Male	21	47.62		Male	17	58.82
Munje	Female	21	61.90	Shidi	Female	13	100.00
	Male	24	66.67		Male	17	64.71
Mwaembe	Female	22	95.45	Stahabu	Female	9	88.89
	Male	20	70.00		Male	15	66.67
Mwandamo	Female	5	80.00				
	Male	32	62.50				

### Kenya

**Fig 3:**
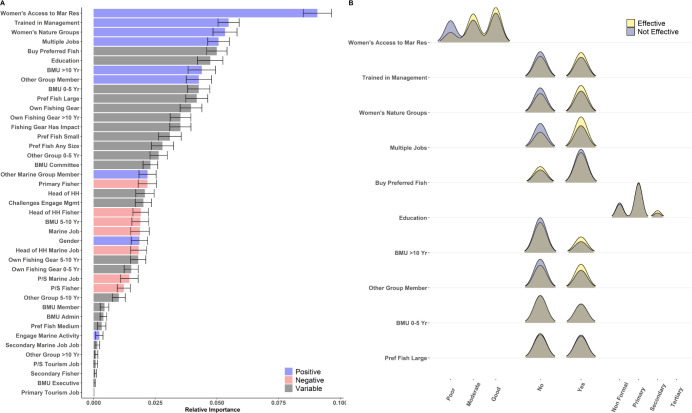
Relative importance and distribution of responses for indicators most strongly associated with differences in perceived management effectiveness in Kenya. (A) Relative importance of features to differences in perceived effectiveness. The length of solid bars along the x-axis represents the average feature importance for each variable across 100 model iterations, and the color of bars indicates relationships between increases in each metric and more positive perceptions of management effectiveness (blue: positive; red: negative; gray: variable). Error bars represent the standard deviation of importance across model runs. (B) Density plots of individual responses for the top 10 most important metrics, separated by individuals that believed management was effective (yellow) and not effective (purple). Brown areas represent overlapping values between individuals that believed management was effective and not effective.

**Fig 4:**
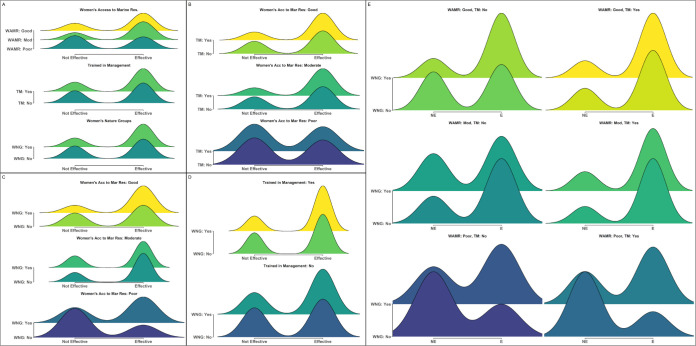
Distributions and interactions between indicators with the strongest relationship to differences in perceived management effectiveness in Kenya. Density plots demonstrating the relative proportion of individuals responding that they believed management was either not effective (left) or effective (right) across different values of: (A) the top three most important indicators (WAMR: Women’s Access to Marine Resources; TM: Trained in Management; WNG: Women’s Nature Groups); (B-D) two-way interactions between the top three indicators; and (E) three-way interactions between all indicators (x-axis: (NE) not effective; (E) effective). Numbers on either side of density plots represent the total number of individuals for each feature or combination of features that believed management was effective (right side) or not effective (left side).

Responses of “effective” and “not effective” in the Kenya test data were predicted with an accuracy of 68.2 ± 2.5% across all 100 model runs (Fig 3). The three most important features selected by model iterations were all associated with increased likelihood of perceived effectiveness: increased access to marine resources for women, whether individuals had received training on best practices in marine resource management, and the presence of women’s groups engaged in nature-based enterprises. When respondents indicated that “women’s access to fish and marine resources” was poor, only 48% (n = 65) of respondents believed that management was effective, compared to 72% (n = 123) and 70% (n = 154) when respondents indicated that access was moderate or good, respectively (Fig 4A). These patterns were consistent across genders, with women and men demonstrating similar increases in rates of perceived effectiveness when they felt women’s access to marine resources was poor (women: 55% [n = 29]; men: 42% [n = 36]), moderate (women: 80% [n = 51]; men: 65% [n = 72]), and good (women: 79% [n = 54]; men: 65% [n = 100]). Similarly, the presence of women’s groups engaged in nature-based enterprises increased perceived management effectiveness from 61% (n = 158) to 72% (n = 191), and again increased positive perceptions across both genders (women: from 70% [n = 66] to 81% [n = 72]; men: from 54% [n = 92] to 66% [n = 119]). Finally, individuals that indicated they had not received training in best practices for marine resource management believed management was effective only 62% (n = 171) of the time, compared to 71% (n = 176) of individuals that had received management training.

Even stronger patterns were found when assessing two- and three-way interactions between features (Fig 4B-4E). For example, when individuals indicated that women’s access to marine resources was poor and they had not received training on management best practices, only 48% (n = 25) believed that management was effective (Fig 4B). In contrast, when individuals indicated that women’s access to marine resources was good and they had received management training, 77% (n = 69) of respondents indicated that management was effective. Similar synergistic interactions were seen between women’s access to marine resources and the presence of women’s groups engaged in nature-based enterprises (Fig 4C), having management training and the presence of women’s groups engaged in nature-based enterprises (Fig 4D), and interactions between all three features (Fig 4E).

### Tanzania

**Fig 5:**
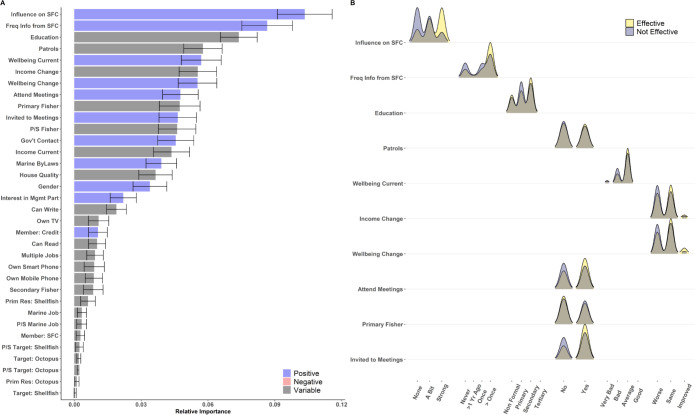
Relative importance and distribution of responses for indicators most strongly associated with differences in perceived management effectiveness in Tanzania. (A) Relative importance of features to differences in perceived effectiveness. The length of solid bars along the x-axis represents the average feature importance for each variable across 100 model iterations, and the color of bars indicates relationships between increases in each metric and more positive perceptions of management effectiveness (blue: positive; red: negative; gray: variable). Error bars represent the standard deviation of importance across model runs. (B) Density plots of individual responses for the top 10 most important metrics, separated by individuals that believed management was effective (yellow) and not effective (purple). Brown areas represent overlapping values between individuals that believed management was effective and not effective.

**Fig 6:**
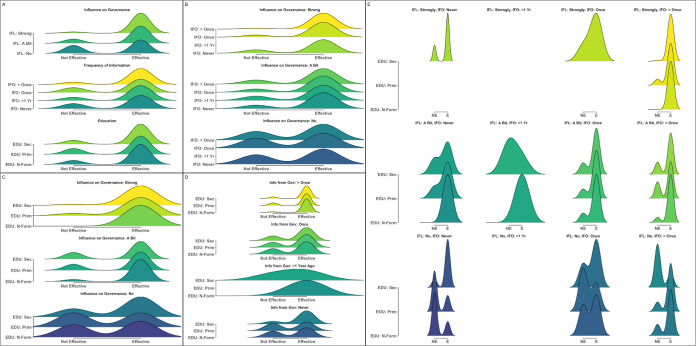
Distributions and interactions between indicators with the strongest relationship to differences in perceived management effectiveness in Tanzania. Density plots demonstrating the relative proportion of individuals responding that they believed management was either not effective (left) or effective (right) across different values of: (A) the top three most important indicators (IFL: Influence on Governance; IFO: Frequency of Information from Governing Bodies; EDU: Education Level); (B-D) two-way interactions between the top three indicators; and (E) three-way interactions between all indicators (x-axis: (NE) not effective; (E) effective). Numbers on either side of density plots represent the total number of individuals for each feature or combination of features that believed management was effective (right side) or not effective (left side).

Models for Tanzania accurately predicted “effective” and “not effective” responses in the test data 76.8 ± 2.5% of the time (Fig 5). The two most important features found across model iterations were again associated with an increased probability of perceived effectiveness: the level of influence individuals felt they had on governance and an increased frequency of information coming from management bodies. The third most important predictor—individuals’ highest attained education level—was generally positively related to perceptions of management, although some variation did exist. When individuals felt that they did not have influence on governing bodies, only 62% (n = 64) of respondents felt that management was effective, compared to 80% (n = 91) and 94% (n = 113) when individuals felt that they had “a bit” of influence or strong influence, respectively (Fig 6A). Similarly, only 72% (n = 50) of individuals who had never received information from governing bodies believed that management was effective, compared to 87% (n = 170) of individuals who had received information more than once in the preceding year. Finally, the education level of respondents was found to be important in model predictions, but had slightly variable impacts on perceived management effectiveness (non-formal education: 83% effective (n = 60); primary education: 76% (n = 82); secondary education: 84% (n = 117); tertiary education: 100% (n = 3)).

Two- and three-way interactions between features again produced even stronger trends in perceived effectiveness (Fig 6B-6E). When individuals indicated that they had no influence on governance and had never received information from governing bodies, only 62% (n = 26) believed that management was effective (Fig 6B). In contrast, when individuals felt they had strong influence on local governance and had received information from governing bodies more than once in the past year, 95% (n = 99) of respondents believed that management was effective. Increases in perceived effectiveness were also observed for simultaneous increases in perceived influence on governance and the education level (Fig 6C) as well as increased frequency of information from governing bodies and education level (Fig 6D), as well as interactions between all three features (Fig 6E).

## Discussion

In both Kenya and Tanzania, community perceptions of CBC management effectiveness were generally positive, and could be predicted with relatively high accuracy using quantifiable metrics, indicating that individual perceptions were linked to local conditions rather than stochastic processes. In addition, when individuals responded favorably to multiple indicators, synergistic effects produced even higher rates of perceived management effectiveness. Most interestingly, despite disparities in the indicators measured by survey instruments in each country, the most powerful metrics for predicting differences between individuals in both Kenya and Tanzania were strongly related to community and individual empowerment in marine resource management. In particular, improved perceptions of management effectiveness in Kenya were strongly associated with women’s empowerment in CBC programs, while in Tanzania the strongest predictors of perceived management effectiveness involved the existence of transparent and participatory processes that allowed individuals to influence marine resource governance.

Further, connections between perceptions of local empowerment and management effectiveness were not primarily impacted by whether individuals were members of BMUs or SFCs or whether individuals were fishers or employed in other marine-based occupations, as most metrics related to these factors had low predictive power in both models. This suggests that the perceived ability to influence management was the predominant driver of differential perceptions of effectiveness, rather than cognitive biases (e.g., in-group favoritism) promoting positive perceptions of management effectiveness through membership in governing bodies alone. These results highlight the necessity of CBC initiatives to not only ensure that local communities have the capacity to actively engage in resource management, but also take into account local context to understand what different resource users value as the most important metrics of CBC success.

### Kenya

In Kenya, more positive perceptions of CBC effectiveness were strongly associated with increased gender equity, both in terms of women’s access to marine resources and the existence of women’s groups engaged in nature-based enterprises. Although CBC participation and management in Kenya has often been male dominated [41, 42], there have been multiple national-level initiatives aimed at improving gender equality throughout Kenyan society. These have included ratifying international commitments such as the Convention on the Elimination of all forms of Discrimination Against Women and Sustainable Development Goals, as well as the establishment of the National Gender and Equality Commission and State Department of Gender within the national government. In addition, recent NGO-led initiatives have worked to increase women’s membership and participation in BMUs, thereby giving them access to high-quality fish. These programs also provided access to refrigeration and supported the transformation of group savings schemes into more sustainable village savings and loans programs that allowed women to grow individual businesses. It is possible that these initiatives resulted in more widespread knowledge of the benefits of gender equality within CBC programs and society at large, and could therefore have made increased gender equity one of the most important markers of CBC “success” within communities.

Similarly, more favorable perceptions of governance effectiveness when women’s groups engaged in nature-based enterprises were present may be related to increased economic opportunity these groups can provide to communities. For example, the Bidii Women Group in Mwaembe, found in one of the surveyed sites, established a group fish depot that allows them to buy and sell fish collaboratively. By pooling financial resources, the group can access fish from distant depots during the low season when local fish are less readily available. Other groups, such as the Tunusuru Conservation Women Group in Munje, focus on providing alternative nature-based income sources beyond fisheries, including planting and selling mangrove seedlings for restoration and a bee-keeping program that operates adjacent to mangrove restoration sites, both of which help support and finance one another. The function that these groups play in providing complementary revenue streams to women in coastal communities is closely tied to the livelihood security of coastal communities in general (e.g., [43]), which may explain the strong relationship between perceptions of management effectiveness and the presence of women’s groups engaged in nature-based enterprises.

There is also substantial evidence that suggests that increasing the participation of women in CBC programs leads to realized conservation benefits within communities. Women’s empowerment has been linked to increased agricultural yields and livelihood diversification in Kenya [44, 45] improved forest biomass and forest-related incomes in India [46], and more positive outcomes for forestry and fishery governance across multiple sites in India and Nepal [47]. Mixed-gender groups have also been demonstrated to reach more socially optimal outcomes in experimental games regarding decisions around contributions and extractions from common-pool resources in Kenya [48], and the presence of women has been shown to increase collaboration, conflict resolution, and capacity for collective action in natural resource management groups across multiple contexts [49]. In Tanzania, female fisheries collectives have been demonstrated to improve female fishers’ livelihoods [50], which can improve community-level financial resiliency and ability to support local development projects [51]. These findings underscore the importance of explicitly incorporating mechanisms that enhance gender equity within management systems and participatory processes. While overcoming systemic gender inequalities will be difficult and context-specific, an increasing number of frameworks and recommendations for gender transformative change exist, including for marine systems (e.g., [52]).

Improved perceptions of CBC effectiveness were also associated with individuals that indicated they had received training on “best practices” in marine resource management, primarily led by local NGOs or government agencies such as the Kenya Marine and Fisheries Research Institute. The topics that these trainings covered varied between respondents, but most often included general conservation principles (such as ecological processes, mangrove and coral restoration, and protected areas), the use of non-destructive fishing gears, halting the collection of juvenile fishes, as well as improving the business and financial literacy of fishers. Greater knowledge of ecosystem functioning has previously been demonstrated to increase positive attitudes towards natural resource management across multiple contexts [53, 54], as such training can reduce misconceptions about the intentions of conservation initiatives and improve knowledge of both potential and realized community-level benefits. Similarly, specific training focusing on the impacts of destructive fishing gears and harvesting of juvenile fish may have helped alleviate concerns over costs such as the potential for lost income caused by changes in gear and fish selection through improved understanding of long-term ecological and financial benefits of CBC management.

Only three other highly important predictors had consistent directional associations with patterns of perceived management effectiveness, all of which were positive: whether individuals reported having multiple jobs; whether individuals had been members of their local BMU for over 10 years; and whether individuals reported being members of local organizations that were not the BMU. The presence of multiple livelihood sources may be a direct result of resource management actions within these communities, as promoting sustainable, non-extractive livelihoods is one of the primary functions of local BMUs. In addition, multiple income streams may increase access to fish and other marine resources, or lower dependence on such resources, which may also improve individual perceptions of resource management actions [55]. More positive perceptions of individuals that have been active BMU members for long periods may be due to better understanding of the intentions and reasoning behind management rules or more direct influence on regulations and therefore increased feelings of ownership of management [56]. Additionally, those with increased knowledge of long-term ecological trends may have lower susceptibility to “shifted baselines” when assessing the necessity of conservation actions. However, it is also possible that individuals that have been BMU members for extended periods are more prone to “sunk cost” or “status quo” biases that would make them less likely to advocate for changes to resource management. Membership in other local organizations may improve perceptions of management through increased connections within the community, which can provide greater opportunities to participate in decision-making [57] and higher levels of trust and accountability, both of which are critical for effective collective action [58].

Conversely, almost all of the metrics associated with more negative perceptions of management effectiveness were related to fishing or other marine activities being a primary source of household income. This pattern is not surprising, given that these individuals are the most likely to be immediately and directly impacted by conservation measures, particularly early after their establishment. However, it is interesting to note that these metrics had relatively low predictive power, indicating that additional training and highlighting the ecological and economic benefits of management, coupled with adequate support for sustainable resource-use capacity, may be able to overcome negative perceptions that may initially be held by fishers.

### Tanzania

In Tanzania, two of the top three indicators associated with improved perceptions of CBC effectiveness were related to the existence of participatory and transparent governance structures within management regimes. First, respondents that felt they had the ability to influence the decisions made by their SFC were considerably more likely to perceive management as being effective, highlighting the importance of collective-choice arrangements within CBC initiatives [8]. This finding is congruent with previous research demonstrating that when individuals that are affected by management rules can participate in creating and modifying those rules, local structures and regulations are more likely to be perceived as legitimate by resource users [59]. In addition, devolving decision-making processes to local levels can ensure that rules are congruent with local social and ecological conditions and that the relative costs and benefits of regulations are proportional between different user groups [8], which has been shown to be critical to CBC programs across multiple contexts [60].

Second, perceptions of management effectiveness improved with the frequency of information individuals received from governing bodies. Communication between management authorities and resource users is critical to ensure that those responsible for monitoring and enforcement can be held accountable by resource users [8], making transparent decision-making a prerequisite for equitable management [61, 62]. Thus, the amount and quality of information coming from governing bodies likely impacted individuals’ ability to influence governance and, therefore, perceptions of overall management effectiveness. In addition to these metrics, several other indicators that demonstrated both strong predictive power and directional effects were also related to participatory processes and transparent governance. Respondents were considerably more likely to report that management was effective if they had been invited to meetings to discuss marine resource use, had attended such meetings, or if they indicated that representatives from the Zanzibar Department of Fisheries Development had come to the community to discuss resource management.

These results reinforce previous findings demonstrating that CBC programs that effectively devolve decision-making authority to local resource users can not only achieve more positive ecological outcomes [22], but also promote greater support for conservation programs among local resource users [45]. To maintain long-term success and support from resource users, CBC programs should therefore: (a) prioritize the co-design of governance that is led and managed by resource users over “consultative” co-management where decision-making authority ultimately lies solely with governments [63]; (b) incorporate or strengthen structures that promote inclusive and participatory decision-making processes and local tenure rights [64, 65]; (c) incorporate customary management into CBC initiatives where appropriate [66]; and (d) ensure *downward* accountability (from those with decision-making authority down to constituents) through mechanisms such as audits and evaluations, explicit public reporting guidelines, and procedures for recall and referendums [67].

To this end, NGOs in the region have been working with SFCs to implement “Self-Assessment” tools that allow governing bodies (and often their constituents) to reflect on the current status of management effectiveness and prioritize future actions. These tools focus on a variety of governance characteristics, including indicators of transparency and communication with communities, management plans and implementation, financial plans and audits, and participatory processes including gender representation in management. Self-Assessment tools can therefore highlight whether communication between SFCs and local resource-users (often conducted through village-level meetings) occurs with enough frequency to ensure that stakeholders are adequately informed of SFC mandates and actions, and whether resource users are currently given sufficient opportunity to share information and discuss issues with SFC members to ultimately have their needs incorporated into management actions. Given the high importance resource users placed on participatory and transparent governance processes, broader use of these tools could greatly improve the perceived legitimacy and efficacy of CBC programs throughout the region.

More positive perceptions of CBC efficacy were also observed with increasing levels of formal education of respondents. There are many potential reasons for this association. Previous investigations have demonstrated that higher education levels can increase participation in environmental management [68], improving trust and equity between users and the formal institutions governing resources [59]. Higher education levels have also been demonstrated to increase risk aversion in pastoral women when assessing environmental challenges and thereby improve overall adaptive capacity [69]. More broadly, individuals with greater formal education have been shown to be more aware of environmental issues and exhibit overall greater support for conservation across multiple regions including Kenya [70], India [71], Uganda [72], and Madagascar [73]. However, individuals with higher education in these studies were often employed in higher-income sectors and had less direct reliance on the natural resources being conserved, and likely less direct engagement with resource management. The majority of individuals surveyed in this study did not attain higher than a primary level of education, and although self-reported levels of current income and changes in income over the past year were relatively important predictors of perceived management effectiveness, neither had strong directional effects on changes in perceptions. However, both improved current wellbeing and wellbeing trends over the past year were associated with higher likelihoods of individuals indicating that they believed CBC management was effective, which may be representative of similar processes.

## Conclusions

Individuals’ perceptions of CBC management effectiveness in both Kenya and Tanzania were strongly related to individual and community empowerment in CBC management, although through different focal areas within each country. That indicators related to these domains—women’s empowerment in Kenya and participatory and transparent governance in Tanzania—were consistently linked to improved perceptions of CBC effectiveness can help NGOs and other organizations supporting CBC in the region design and implement programs that can achieve ecological objectives while also satisfying the most critical needs and desires of community members. Tailoring current and future CBC initiatives to emphasize processes and outcomes most closely linked to these community priorities can help foster broader support for conservation programs and overcome resistance to regulations by highlighting the benefits provided by effective CBC management.

Findings from this study provide additional examples that underscore more general trends that have long been recognized in CBC research and practice communities: CBC is context-specific, and while we illustrate here important relationships between perceptions of management effectiveness and key governance principles, neither survey instrument used in Kenya or Tanzania encapsulated all possible drivers of differences in perceptions between individuals. There are many other factors that could potentially influence individual and group perceptions of management in both countries, including those that were strong predictors in one country but not assessed in the other. Future research could explore these factors in more depth. This study was also unable to examine the relationships between perceptions of management effectiveness and realized (rather than perceived) ecological and socio-economic outcomes of CBC programs or whether differences in individuals’ unique management goals or metrics of “success” impacted their perceptions of CBC efficacy. Future investigations examining these associations will be necessary to understand the relative impact of the metrics most often used to define CBC success by implementing agencies on the continued support of CBC programs from local communities.

Despite these limitations, our results provide a foundational understanding of what influences differential perceptions of CBC effectiveness in some parts of coastal Kenya and Tanzania that can be utilized to help promote community support for conservation and be refined through additional quantitative and qualitative investigations. As CBC programs continue to expand as primary tools for both environmental conservation, social, and economic development, understanding the underlying drivers of community support for such programs will be critical for their long-term sustainability. While achieving joint and reinforcing social-ecological benefits is the central goal of CBC initiatives, how “success” is defined by local resource users, and what indicators or benchmarks will most readily and effectively indicate such success, is likely to vary across contexts. Continued support for CBC programs will be dependent upon resource users’ perceptions of management effectiveness, which will require CBC initiatives to not only develop management regimes that meet community needs, but also clearly demonstrate that outcomes that matter most to the communities that they impact are realized.

## Supporting information

S1 Appendix Predictive metrics used in xgboost classification models for Kenya and Tanzania. (DOCX)
