# Supplementary material for: Individual and community empowerment improve resource users’ perceptions of community-based conservation effectiveness in Kenya and Tanzania
Source: PLoS One. 2024 Apr 30;19(4):e0301345. doi: 10.1371/journal.pone.0301345 (PMC11060543; doi:10.1371/journal.pone.0301345)
Supplement: S1 Appendix — (DOCX) [file pone.0301345.s001.docx]

**S1 Appendix: Predictive metrics used in xgboost classification models for Kenya and Tanzania.**

**Table S1.** Predictive metrics used in xgboost classification models for Kenya, as well as distribution of responses for each value.

| Category | Feature | Description |  |  |  |  |  |  |
| --- | --- | --- | --- | --- | --- | --- | --- | --- |
| Demographic Characteristics | Gender | Gender of respondent | Value | 1 (Male) | 2 (Female) | No Answer |  |  |
|  |  |  | n | 211 | 138 | 0 |  |  |
|  | Education | Highest education level | Value | 1  (Non-Formal) | 2  (Primary) | 3 (Secondary) | 4  (Tertiary) | No Answer |
|  |  |  | n | 88 | 218 | 40 | 2 | 1 |
|  | Head of HH | Whether respondent is the head of the household | Value | 0 (No) | 1 (Yes) | No Answer |  |  |
|  |  |  | n | 71 | 278 | 0 |  |  |
| Membership in Local Marine Governance | BMU Member | Member of the BMU | Value | 0 (No) | 1 (Yes) | No Answer |  |  |
|  |  |  | n | 22 | 327 | 0 |  |  |
|  | BMU 0-5 Yr | Member of the BMU for 0-5 years | Value | 0 (No) | 1 (Yes) | No Answer |  |  |
|  |  |  | n | 204 | 141 | 4 |  |  |
|  | BMU 5-10 Yr | Member of the BMU for 5-10 years | Value | 0 (No) | 1 (Yes) | No Answer |  |  |
|  |  |  | n | 280 | 65 | 4 |  |  |
|  | BMU >10 Yr | Member of the BMU for longer than 10 years | Value | 0 (No) | 1 (Yes) | No Answer |  |  |
|  |  |  | n | 238 | 107 | 4 |  |  |
|  | BMU Admin | Administrative member of the BMU | Value | 0 (No) | 1 (Yes) | No Answer |  |  |
|  |  |  | n | 326 | 23 | 0 |  |  |
|  | BMU Committee | Committee member of the BMU | Value | 0 (No) | 1 (Yes) | No Answer |  |  |
|  |  |  | n | 312 | 37 | 0 |  |  |
|  | BMU Executive | Executive member of the BMU | Value | 0 (No) | 1 (Yes) | No Answer |  |  |
|  |  |  | n | 344 | 5 | 0 |  |  |

| Membership in Other Local Organizations | Other Group Member | Whether the respondent is a member of another local group | Value | 0 (No) | 1 (Yes) | No Answer |  |  |
| --- | --- | --- | --- | --- | --- | --- | --- | --- |
|  |  |  | n | 186 | 163 | 0 |  |  |
|  | Other Marine Group Member | Whether the respondent is a member of another marine-related local group | Value | 0 (No) | 1 (Yes) | No Answer |  |  |
|  |  |  | n | 271 | 78 | 0 |  |  |
|  | Other Group 0-5 Yr | Whether the respondent has been a member of another local group for 0-5 years | Value | 0 (No) | 1 (Yes) | No Answer |  |  |
|  |  |  | n | 255 | 82 | 12 |  |  |
|  | Other Group 5-10 Yr | Whether the respondent has been a member of another local group for5-10 years | Value | 0 (No) | 1 (Yes) | No Answer |  |  |
|  |  |  | n | 306 | 31 | 12 |  |  |
|  | Other Group >10 Yr | Whether the respondent has been a member of another local group for more than 10 years | Value | 0 (No) | 1 (Yes) | No Answer |  |  |
|  |  |  | n | 328 | 9 | 12 |  |  |
| Women's Participation in Marine Resources and Management | Women's Access to Mar Res | Respondent's opinion of the ability of women to access fish and other marine resources | Value | 1 (Poor) | 2 (Moderate) | 3 (Good) | No Answer |  |
|  |  |  | n | 65 | 123 | 154 | 7 |  |
|  | Women's Nature Groups | Whether the respondent is aware of women's groups participating in nature based enterprises | Value | 0 (No) | 1 (Yes) | No Answer |  |  |
|  |  |  | n | 158 | 191 | 0 |  |  |
| Livelihoods: Head of Household | Head of HH Fisher | Whether the head of the household is a fisher | Value | 0 (No) | 1 (Yes) | No Answer |  |  |
|  |  |  | n | 183 | 166 | 0 |  |  |
|  | Head of HH Marine Job | Whether the head of the household works in a marine-related job | Value | 0 (No) | 1 (Yes) | No Answer |  |  |
|  |  |  | n | 148 | 201 | 0 |  |  |
|  | Head of HH Tourism Job | Whether the head of the household works in a tourism-related job | Value | 0 (No) | 1 (Yes) | No Answer |  |  |
|  |  |  | n | 347 | 2 | 0 |  |  |
|  | Head of HH Unemployment | Whether the head of the household is unemployed |  | 0 (No) | 1 (Yes) | No Answer |  |  |
|  |  |  | n | 348 | 1 | 0 |  |  |

| Livelihoods: Respondent | Primary Fisher | Whether respondent indicated fisher as their primary occupation | Value | 0 (No) | 1 (Yes) | No Answer |  |  |
| --- | --- | --- | --- | --- | --- | --- | --- | --- |
|  |  |  | n | 173 | 176 | 0 |  |  |
|  | Marine Job | Whether respondent indicated a marine-related job as their primary occupation | Value | 0 (No) | 1 (Yes) | No Answer |  |  |
|  |  |  | n | 132 | 217 | 0 |  |  |
|  | Primary Tourism Job | Whether respondent indicated tourism as their primary occupation | Value | 0 (No) | 1 (Yes) | No Answer |  |  |
|  |  |  | n | 342 | 7 | 0 |  |  |
|  | Secondary Fisher | Whether respondent indicated fisher as their secondary occupation | Value | 0 (No) | 1 (Yes) | No Answer |  |  |
|  |  |  | n | 340 | 9 | 0 |  |  |
|  | Secondary Marine Job | Whether respondent indicated a marine-related job as their secondary occupation | Value | 0 (No) | 1 (Yes) | No Answer |  |  |
|  |  |  | n | 328 | 21 | 0 |  |  |
|  | Secondary Tourism Job | Whether respondent indicated tourism as their secondary occupation | Value | 0 (No) | 1 (Yes) | No Answer |  |  |
|  |  |  | n | 346 | 3 | 0 |  |  |
|  | P/S Fisher | Whether the respondent indicated fisher as their primary or secondary occupation | Value | 0 (No) | 1 (Yes) | No Answer |  |  |
|  |  |  | n | 166 | 183 | 0 |  |  |
|  | P/S Marine Job | Whether the respondent indicated a marine-related job as their primary or secondary occupation | Value | 0 (No) | 1 (Yes) | No Answer |  |  |
|  |  |  | n | 119 | 230 | 0 |  |  |
|  | P/S Tourism Job | Whether the respondent indicated tourism as their primary or secondary occupation | Value | 0 (No) | 1 (Yes) | No Answer |  |  |
|  |  |  | n | 339 | 10 | 0 |  |  |
|  | Multiple Jobs | Whether the respondent has multiple jobs | Value | 0 (No) | 1 (Yes) | No Answer |  |  |
|  |  |  | n | 139 | 210 | 0 |  |  |
|  | Unemployment | Whether the respondent is unemployed | Value | 0 (No) | 1 (Yes) | No Answer |  |  |
|  |  |  | n | 348 | 1 | 0 |  |  |

| Fishing Gear Ownership and Impacts | Own Fishing Gear | Whether the respondent owns their own fishing gear | Value | 0 (No) | 1 (Yes) | No Answer |  |  |
| --- | --- | --- | --- | --- | --- | --- | --- | --- |
|  |  |  | n | 161 | 188 | 0 |  |  |
|  | Own Fishing Gear >10 Yr | Whether the respondent has owned their own fishing gear for more than 10 years | Value | 0 (No) | 1 (Yes) | No Answer |  |  |
|  |  |  | n | 247 | 96 | 6 |  |  |
|  | Own Fishing Gear 0-5 Yr | Whether the respondent has owned their own fishing gear for 0-5 years | Value | 0 (No) | 1 (Yes) | No Answer |  |  |
|  |  |  | n | 307 | 36 | 6 |  |  |
|  | Own Fishing Gear 5-10 Yr | Whether the respondent has owned their own fishing gear for 5-10 years | Value | 0 (No) | 1 (Yes) | No Answer |  |  |
|  |  |  | n | 293 | 50 | 6 |  |  |
|  | Fishing Gear Has Impact | Whether respondent believes that fishing gear has negative environmental impacts | Value | 0 (No) | 1 (Yes) | No Answer |  |  |
|  |  |  | n | 73 | 257 | 19 |  |  |
| Fish Preferences (Consumption) | Buy Preferred Fish | Whether respondent buys the size of fish they prefer | Value | 0 (No) | 1 (Yes) | No Answer |  |  |
|  |  |  | n | 111 | 231 | 7 |  |  |
|  | Pref Fish Small | Preferred fish: small | Value | 0 (No) | 1 (Yes) | No Answer |  |  |
|  |  |  | n | 249 | 84 | 16 |  |  |
|  | Pref Fish Medium | Preferred fish: medium | Value | 0 (No) | 1 (Yes) | No Answer |  |  |
|  |  |  | n | 314 | 19 | 16 |  |  |
|  | Pref Fish Large | Preferred fish: large | Value | 0 (No) | 1 (Yes) | No Answer |  |  |
|  |  |  | n | 173 | 160 | 16 |  |  |
|  | Pref Fish Any Size | Preferred fish: any size | Value | 0 (No) | 1 (Yes) | No Answer |  |  |
|  |  |  | n | 263 | 70 | 16 |  |  |
| Marine Engagement and Management | Engage Marine Activity | Whether the respondent engages in marine or fisheries activities (livelihood or otherwise) | Value | 0 (No) | 1 (Yes) | No Answer |  |  |
|  |  |  | n | 22 | 327 | 0 |  |  |
|  | Challenges Engage Mgmt | Whether respondent has encountered challenges engaging in resource management | Value | 0 (No) | 1 (Yes) | No Answer |  |  |
|  |  |  | n | 58 | 288 | 3 |  |  |
|  | Trained in Management | Whether the individual indicates that they or their group have received training on best practices | Value | 0 (No) | 1 (Yes) | No Answer |  |  |
|  |  |  | n | 171 | 176 | 2 |  |  |

**Table S2.** Predictive metrics used in xgboost classification models for Tanzania, as well as distribution of responses for each value.

| Category | Feature | Description |  |  |  |  |  |  |
| --- | --- | --- | --- | --- | --- | --- | --- | --- |
| Demographics | Gender | Gender of the respondent | Value | 1 (Male) | 2 (Female) | No Answer |  |  |
|  |  |  | n | 162 | 117 | 0 |  |  |
|  | Education | Highest level of education | Value | 1 (Non-Formal) | 2 (Primary) | 3 (Secondary) | 4 (Tertiary) | No Answer |
|  |  |  | n | 60 | 82 | 117 | 3 | 17 |
|  | Can Read | Whether the respondent can read | Value | 0 (No) | 1 (Yes) | No Answer |  |  |
|  |  |  | n | 78 | 201 | 0 |  |  |
|  | Can Write | Whether the respondent can write | Value | 0 (No) | 1 (Yes) | No Answer |  |  |
|  |  |  | n | 85 | 194 | 0 |  |  |
| Engagement with SFC | Interest in Mgmt Part | Whether the respondent is interested in engaging in management | Value | 0 (No) | 1 (Yes) | No Answer |  |  |
|  |  |  | n | 39 | 240 | 0 |  |  |
|  | Invited to Meetings | Whether the respondent has been invited to resource governance meetings | Value | 0 (No) | 1 (Yes) | No Answer |  |  |
|  |  |  | n | 87 | 192 | 0 |  |  |
|  | Attend Meetings | Whether the respondent attends meetings of the SFC | Value | 0 (No) | 1 (Yes) | No Answer |  |  |
|  |  |  | n | 112 | 167 | 0 |  |  |
|  | Member: SFC | Whether the respondent is a member of the SFC | Value | 0 (No) | 1 (Yes) | No Answer |  |  |
|  |  |  | n | 248 | 31 | 0 |  |  |
|  | Freq Info from SFC | How often the respondent reported receiving information from the local SFC in the past year | Value | 1 (Never) | 2 (> 1 Year Ago) | 2 (Once) | 3 (> Once) | No Answer |
|  |  |  | n | 50 | 11 | 48 | 170 | 0 |
|  | Influence on SFC | The level the respondent feels they are able to influence the SFC | Value | 1 (None) | 2 (A Bit) | 3 (Strong) | No Answer |  |
|  |  |  | n | 64 | 91 | 113 | 11 |  |

| Management | Marine ByLaws | Whether the respondent indicates that there are marine bylaws or management in place | Value | 0 (No) | 1 (Yes) | No Answer |  |  |
| --- | --- | --- | --- | --- | --- | --- | --- | --- |
|  |  |  | n | 22 | 239 | 18 |  |  |
|  | Gov't Contact | Whether anyone from DFD or DFO has come to the community to speak about management | Value | 0 (No) | 1 (Yes) | No Answer |  |  |
|  |  |  | n | 82 | 197 | 0 |  |  |
|  | Patrols | Whether the respondent reports having seen DFD rangers patrolling their fishing grounds | Value | 0 (No) | 1 (Yes) | No Answer |  |  |
|  |  |  | n | 144 | 135 | 0 |  |  |
| Wellbeing and Assets | Wellbeing Current | Respondents reported current wellbeing | Value | 1 (Very Bad) | 2 (Bad) | 3 (Average) | 4 (Good) | No Answer |
|  |  |  | n | 13 | 62 | 199 | 5 | 0 |
|  | Wellbeing Change | Respondent's reported change in wellbeing since the previous year | Value | 1 (Worse) | 2 (Same) | 3 (Improved) | No Answer |  |
|  |  |  | n | 103 | 153 | 23 | 0 |  |
|  | Income Current | Current level of income | Value | 1 (Inadequate) | 2 (Scarce) | 3 (Adequate) | No Answer |  |
|  |  |  | n | 174 | 82 | 20 | 3 |  |
|  | Income Change | Reported change in income from the past year | Value | 1 (Decreased) | 2 (Same) | 3 (Increased) | No Answer |  |
|  |  |  | n | 118 | 142 | 15 | 4 |  |
|  | House Quality | Quality of the respondent's house | Value | 1 (Rudimentary) | 2 (Traditional Stone/Mud) | 3 (Traditional Timber/Metal) | 4 (Modern) | No Answer |
|  |  |  | n | 26 | 31 | 150 | 71 | 1 |
|  | Own Mobile Phone | Whether the respondent reports owning a mobile phone | Value | 0 (No) | 1 (Yes) | No Answer |  |  |
|  |  |  | n | 56 | 223 | 0 |  |  |
|  | Own Smart Phone | Whether the respondent reports owning a smart phone | Value | 0 (No) | 1 (Yes) | No Answer |  |  |
|  |  |  | n | 248 | 31 | 0 |  |  |
|  | Own TV | Whether the respondent reports owning a television | Value | 0 (No) | 1 (Yes) | No Answer |  |  |
|  |  |  | n | 224 | 55 | 0 |  |  |
| Credit Participation | Member: Credit | Whether the respondent is a member of a local credit scheme | Value | 0 (No) | 1 (Yes) | No Answer |  |  |
|  |  |  | n | 203 | 76 | 0 |  |  |

| Livelihoods | Primary Fisher | Whether respondent indicated fisher as their primary occupation | Value | 0 (No) | 1 (Yes) | No Answer |  |  |
| --- | --- | --- | --- | --- | --- | --- | --- | --- |
|  |  |  | n | 136 | 105 | 38 |  |  |
|  | Marine Job | Whether respondent indicated a marine-related job as their primary occupation | Value | 0 (No) | 1 (Yes) | No Answer |  |  |
|  |  |  | n | 172 | 107 | 0 |  |  |
|  | Secondary Fisher | Whether respondent indicated fisher as their secondary occupation | Value | 0 (No) | 1 (Yes) | No Answer |  |  |
|  |  |  | n | 215 | 33 | 31 |  |  |
|  | P/S Fisher | Whether the respondent indicated fisher as their primary or secondary occupation | Value | 0 (No) | 1 (Yes) | No Answer |  |  |
|  |  |  | n | 103 | 125 | 51 |  |  |
|  | P/S Marine Job | Whether the respondent indicated a marine-related job as their primary or secondary occupation | Value | 0 (No) | 1 (Yes) | No Answer |  |  |
|  |  |  | n | 150 | 129 | 0 |  |  |
|  | Multiple Jobs | Whether the respondent has multiple jobs | Value | 0 (No) | 1 (Yes) | No Answer |  |  |
|  |  |  | n | 31 | 248 | 0 |  |  |
| Marine Reliance (Income and Food) | Prim Res: Octopus | Whether the respondent indicates that octopuses are the most important marine resource for the household | Value | 0 (No) | 1 (Yes) | No Answer |  |  |
|  |  |  | n | 259 | 20 | 0 |  |  |
|  | Prim Res: Pelagic Fish | Whether the respondent indicates that pelagic fish are the most important marine resource for the household | Value | 0 (No) | 1 (Yes) | No Answer |  |  |
|  |  |  | n | 247 | 32 | 0 |  |  |
|  | Prim Res: Reef Fish | Whether the respondent indicates that reef fish are the most important marine resource for the household | Value | 0 (No) | 1 (Yes) | No Answer |  |  |
|  |  |  | n | 242 | 37 | 0 |  |  |
|  | Prim Res: Seaweed | Whether the respondent indicates that seaweeds are the most important marine resource for the household | Value | 0 (No) | 1 (Yes) | No Answer |  |  |
|  |  |  | n | 270 | 9 | 0 |  |  |
|  | Prim Res: Shellfish | Whether the respondent indicates that shellfishes are the most important marine resource for the household | Value | 0 (No) | 1 (Yes) | No Answer |  |  |
|  |  |  | n | 255 | 24 | 0 |  |  |
|  | Prim Res: Cucumbers | Whether the respondent indicates that sea cucumbers are the group they target for their livelihood | Value | 0 (No) | 1 (Yes) | No Answer |  |  |
|  |  |  | n | 271 | 8 | 0 |  |  |

| Marine Reliance (Income and Food) | Target: Crabs | Whether the respondent indicates that crabs are the group they target for their livelihood | Value | 0 (No) | 1 (Yes) | No Answer |  |  |
| --- | --- | --- | --- | --- | --- | --- | --- | --- |
|  |  |  | n | 276 | 3 | 0 |  |  |
|  | Target: Octopus | Whether the respondent indicates that octopuses are the group they target for their livelihood | Value | 0 (No) | 1 (Yes) | No Answer |  |  |
|  |  |  | n | 249 | 30 | 0 |  |  |
|  | Target: Pelagic Fish | Whether the respondent indicates that pelagic fish are the group they target for their livelihood | Value | 0 (No) | 1 (Yes) | No Answer |  |  |
|  |  |  | n | 246 | 33 | 0 |  |  |
|  | Target: Reef Fish | Whether the respondent indicates that reef fish are the group they target for their livelihood | Value | 0 (No) | 1 (Yes) | No Answer |  |  |
|  |  |  | n | 234 | 45 | 0 |  |  |
|  | Target: Seaweed | Whether the respondent indicates that seaweeds are the group they target for their livelihood | Value | 0 (No) | 1 (Yes) | No Answer |  |  |
|  |  |  | n | 266 | 13 | 0 |  |  |
|  | Target: Sharks | Whether the respondent indicates that sharks are the group they target for their livelihood | Value | 0 (No) | 1 (Yes) | No Answer |  |  |
|  |  |  | n | 269 | 10 | 0 |  |  |
|  | Target: Shellfish | Whether the respondent indicates that shellfishes are the group they target for their livelihood | Value | 0 (No) | 1 (Yes) | No Answer |  |  |
|  |  |  | n | 240 | 39 | 0 |  |  |
|  | P/S Target: Crabs | Whether the respondent indicates that crabs are the primary or secondary group they target for their livelihood | Value | 0 (No) | 1 (Yes) | No Answer |  |  |
|  |  |  | n | 276 | 3 | 0 |  |  |
|  | P/S Target: Octopus | Whether the respondent indicates that octopuses are the primary or secondary group they target for their livelihood | Value | 0 (No) | 1 (Yes) | No Answer |  |  |
|  |  |  | n | 246 | 33 | 0 |  |  |
|  | P/S Target: Pelagic Fish | Whether the respondent indicates that pelagic fish are the primary or secondary group they target for their livelihood | Value | 0 (No) | 1 (Yes) | No Answer |  |  |
|  |  |  | n | 238 | 41 | 0 |  |  |
|  | P/S Target: Reef Fish | Whether the respondent indicates that reef fish are the primary or secondary group they target for their livelihood | Value | 0 (No) | 1 (Yes) | No Answer |  |  |
|  |  |  | n | 225 | 54 | 0 |  |  |

| Marine Reliance (Income and Food) | P/S Target: Seaweed | Whether the respondent indicates that seaweeds are the primary or secondary group they target for their livelihood | Value | 0 (No) | 1 (Yes) | No Answer |  |  |
| --- | --- | --- | --- | --- | --- | --- | --- | --- |
|  |  |  | n | 263 | 16 | 0 |  |  |
|  | P/S Target: Sharks | Whether the respondent indicates that sharks are the primary or secondary group they target for their livelihood | Value | 0 (No) | 1 (Yes) | No Answer |  |  |
|  |  |  | n | 267 | 12 | 0 |  |  |
|  | P/S Target: Shellfish | Whether the respondent indicates that shellfishes are the primary or secondary group they target for their livelihood | Value | 0 (No) | 1 (Yes) | No Answer |  |  |
|  |  |  | n | 231 | 48 | 0 |  |  |
|  | P/S Target: Cucumbers | Whether the respondent indicates that sea cucumbers are the primary or secondary group they target for their livelihood | Value | 0 (No) | 1 (Yes) | No Answer |  |  |
|  |  |  | n | 271 | 8 | 0 |  |  |
